# Role of ^18^F-FDG PET Scan in Rheumatoid Lung Nodule: Case Report and Review of the Literature

**DOI:** 10.1155/2013/621340

**Published:** 2013-07-25

**Authors:** Christine L. Chhakchhuak, Mehdi Khosravi, Kristine M. Lohr

**Affiliations:** ^1^Division of Rheumatology, Department of Internal Medicine, University of Kentucky, 740 South Limestone Street, Room J-515, Lexington, KY 40536, USA; ^2^Division of Pulmonary and Critical Care Medicine, Department of Internal Medicine, University of Kentucky, 740 South Limestone Street, Kentucky Clinic L 543, Lexington KY 40536, USA

## Abstract

Flourine-18 fluoro-2-deoxy-glucose (^18^F-FDG) positron emission tomography combined with computed tomography (PET/CT) is a useful test for the management of malignant conditions. Inflammatory and infectious processes, however, can cause increased uptake on PET scanning, often causing diagnostic dilemmas. This knowledge is important to the rheumatologist not only because of the inflammatory conditions we treat but also because certain rheumatic diseases impose an increased risk of malignancy either due to the disease itself or as a consequence of medications used to treat the rheumatic diseases. There is an increasing body of evidence investigating the role of PET scans in inflammatory conditions. This paper describes a patient with rheumatoid arthritis who developed pulmonary nodules that showed increased uptake on PET/CT scan and reviews the use of PET scanning in the diagnosis and management of rheumatoid arthritis.

## 1. Introduction

Fluorine-18 fluoro-2-deoxyglucose (^18^F-FDG) positron emission tomography combined with computed tomography (PET/CT) is a useful test to evaluate malignancies [1]. However, inflammatory diseases may also show increased uptake of ^18^F-FDG and cause false-positive PET scan results, necessitating further investigations to rule out malignant conditions [2]. Positron emission tomography (PET) is an analytical imaging technology developed to use compounds labeled with positron-emitting radioisotopes as molecular probes to image and measure biochemical processes in vivo [3]. Numerous tracers have been used in conjunction with PET scanning to aid in the diagnosis of various disorders. ^18^F-FDG has now become the most commonly used radiotracer for PET scanning. Because of the increased metabolic activity of the tumor cells, there is an increased uptake of glucose in tumor cells, thus forming the basis for widespread use of ^18^F-FDG PET scan in the diagnosis, staging, and management of malignancies. However, the increased uptake of ^18^F-FDG tracer is not limited to malignant states and has been seen in benign as well as inflammatory conditions such as sarcoidosis, large vessel vasculitis, inflammatory bowel disease, and rheumatoid arthritis (RA) [2, 4–8]. 

We describe a patient with RA who developed pulmonary nodules, showing increased uptake on ^18^F-FDG PET/CT scan. We also review the current literature on the use of PET scanning in articular and extra-articular RA. 

## 2. Case Report

**Figure 1:**
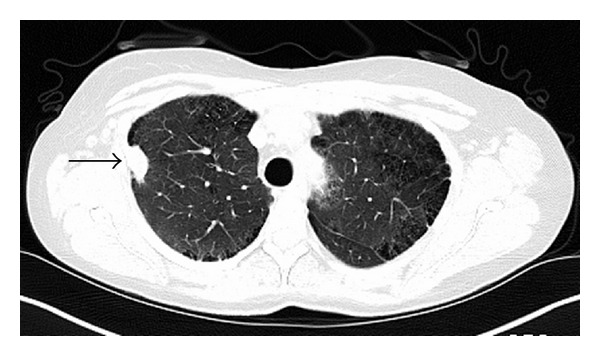
CT chest showing 1.5 cm × 2.5 cm RUL pleural-based nodule (arrow).

**Figure 2:**
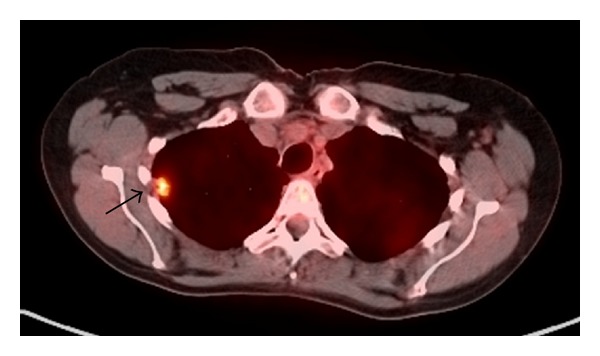
PET scan showing increased uptake in RUL nodule (arrow).

**Figure 3:**
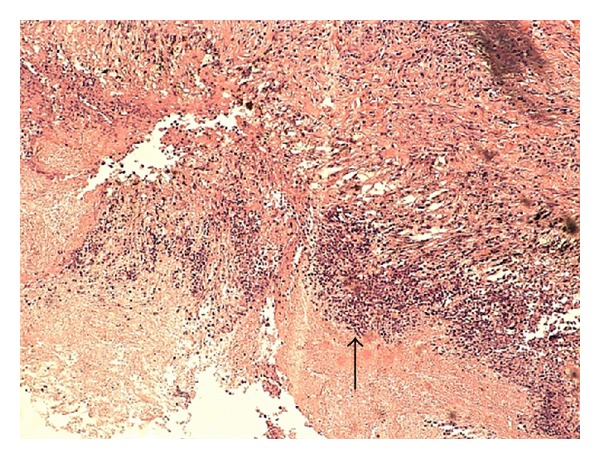
Mixed inflammatory infiltrate seen within the nodule.

**Figure 4:**
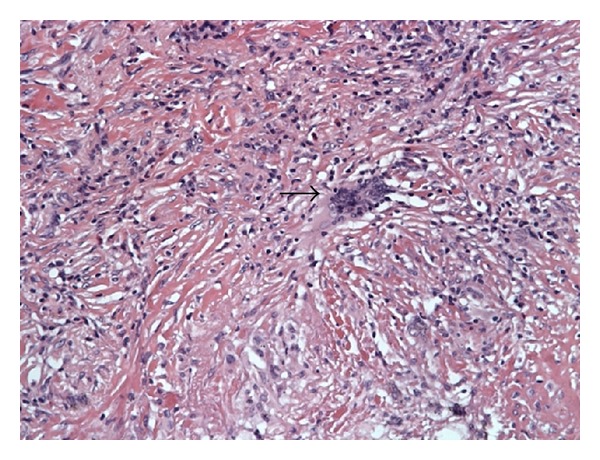
Multinucleated giant cells with lymphocytes, fibroblasts, and collagen in the granuloma wall.

A 50-year-old Caucasian woman with a history of RA presented with dyspnea on exertion. RA was diagnosed 4 years earlier and has been treated with methotrexate since the time of diagnosis. RA is currently in remission on oral methotrexate 15 mg weekly and folic acid 1 mg daily. Both rheumatoid factor and anticitrullinated peptide antibody are highly positive. Past medical history is significant for coronary artery disease with stent placement, chronic obstructive pulmonary disease (COPD), hypothyroidism, hypertension, and gastroesophageal reflux disease. She has a 60-pack-year smoking history and continues to smoke. Family history is positive for a mother and sister with lung cancer who were both heavy smokers and 2 sisters with COPD. She has lost 15–20 pounds of weight intentionally over a period of 3 months. There is history of loss of appetite. Physical examination including joint examination is normal except for decreased breath sounds in both upper lobes. Laboratory data revealed normal blood count, erythrocyte sedimentation rate (ESR), C-reactive protein (CRP), and renal and liver functions. A chest X-ray done for evaluation of dyspnea on exertion revealed a new right upper lobe (RUL) lung nodule. Her previous chest X-ray four years earlier showed multiple calcified nodules bilaterally and a small noncalcified RUL nodule less than a centimeter. CT chest revealed a 1.5 cm × 2.5 cm noncalcified pleural-based RUL nodule, a 1.1 cm calcified nodule in the left upper lobe, and an 8 mm noncalcified nodule in the right lower lobe (Figure 1). ^18^F-FDG PET/CT scan showed moderately increased uptake in all nodules with a maximum standardized uptake value (SUV) of 3.7 in the RUL nodule (Figure 2). Given the patient's high risk based on tobacco use and family history of lung cancer, she underwent a CT-guided biopsy of the RUL nodule that was nondiagnostic. Subsequent video-assisted thoracic surgery (VATS) biopsy of the right upper and lower lobe nodules was performed. Pathology of both nodules revealed chronic inflammation with necrotizing granulomatous formation consistent with rheumatoid nodules and no evidence of malignancy (Figures 3 and 4). Fungal and acid-fast bacilli stains and cultures were negative. 

## 3. Discussion

It has been noted that around 50 percent of solitary pulmonary nodules and a greater percentage of multiple pulmonary nodules turn out to be benign processes [9]. This, in the face of 200,000 cases of lung cancer diagnosed per year with mortality of 150,000 per year, creates a difficult conundrum in the management of pulmonary nodules in diseases known to cause lung nodules such as RA [10]. ^18^F-FDG PET/CT scan has a sensitivity of 96-97% and a specificity of 83–85% in differentiating malignant pulmonary nodules [11–13]. However, the presence of inflammation and fibrosis significantly hinders PET scan accuracy. This is compromised even more so in the era of RA treatment with biologics that confers increased risk of mycobacterial and fungal infections which can potentially present as PET-positive lesions.

Reports of the use of PET and PET/CT in extra-articular RA are limited to subcutaneous nodules, lymph nodes, and the lung. Rodríguez et al. described two patients with RA in whom pulmonary nodules showed increased SUV on ^18^F-FDG PET scan [14]. Biopsy of the nodules demonstrated bronchogenic carcinoma developing within preexisting rheumatoid nodules. Based on their experience they concluded that PET scan is a diagnostic test of high accuracy and can be used before surgical biopsy in patients with RA and pulmonary nodules who are suspected to have bronchogenic carcinoma. However, not all cases of increased uptake on PET scan are related to malignancy as is evidenced by the following reports. Gupta et al. described a patient with RA found to have mild increased uptake in pulmonary nodules on PET scan [15]. Histological examination of these nodules revealed the presence of rheumatoid nodules. Bagga reported a case of a 63-year-old female with severe RA and long-standing smoking history [16]. The patient had multiple pulmonary nodules and a left pleural effusion. PET scan showed no uptake in the lung nodules but intense pleural uptake. A CT-guided biopsy of both the nodules and the pleura was performed, which confirmed the diagnosis of rheumatoid lung disease. dos Anjos et al. reported a patient with RA in whom increased ^18^F-FDG uptake was found in subcutaneous nodules and lymph nodes including cervical, supraclavicular, axillary, and pelvic areas [17]. Similarly, Seldin et al. have published a series of nine patients with RA having increased FDG tracer uptake in the axillary lymph nodes [18]. Strobel et al. described a patient with RA with a soft tissue nodule on the left elbow [19]. Based on moderately increased activity on PET scan, the patient underwent excision of the nodule. It turned out to be a rheumatoid nodule.

## 4. Conclusion

The role of PET scan in diagnosis, staging, and management of malignancies is well known. There is an increasing body of evidence supporting the role of PET scans in nonmalignant conditions including vasculitis and granulomatous diseases such as sarcoidosis [20, 21]. The major factors limiting widespread use of PET/CT scanning appear to be cost, availability, and radiation exposure. The role of PET scan in cases of extra-articular involvement of RA has not been studied and is limited to a very small number of case reports and case series. 

As evidenced by our case as well as other case reports, increased activity on PET scan does not necessarily translate into the diagnosis of malignancy. One would generally assume that the degree of maximum FDG uptake is lower in inflammatory lung nodules compared to malignancies. However, this is not always the case, as RA nodules have been reported with SUVs ranging from low to very high [15, 16, 19, 22, 23]. Thus, SUV cannot be used to differentiate between benign inflammatory and malignant lesions. Serial monitoring with PET/CT scans may show a more stable SUV uptake in benign inflammatory lesions compared to malignancies. Recently, the use of 11C-methionine PET scan has been shown to be more sensitive in differentiating between malignant and inflammatory lung lesions [24, 25]. ^18^F-FDG PET scan provides valuable information on joint involvement as well as extra-articular disease in patients with RA. As promising as PET scanning is for inflammatory joint conditions, larger studies are necessary, especially pertaining to the cost effectiveness of routine PET scanning before its use in clinical practice. Clinicians need to be aware of the fact that rheumatoid nodules can have increased activity on PET scan. With the increasing use of PET scan for evaluation of lung nodules, rheumatologists will likely be confronted with more such cases of increased PET uptake in RA patients. Considering the lack of an established range of rheumatoid nodule metabolic activity, close monitoring, with or without the use of invasive diagnostic methods such as needle biopsy or VATS, is prudent in the management of lung nodules in rheumatoid arthritis.
